# Supplementary material for: microRNA arm-imbalance in part from complementary targets mediated decay promotes gastric cancer progression
Source: Nat Commun. 2019 Sep 27;10:4397. doi: 10.1038/s41467-019-12292-5 (PMC6764945; doi:10.1038/s41467-019-12292-5)
Supplement: Supplementary file 1 — Supplementary Information [file 41467_2019_12292_MOESM1_ESM.pdf]

Supplementary Information

microRNA arm-imbalance partially from targets-mediated decay promotes gastric cancer progression

Zhang *et al*. 2019

Supplementary figures

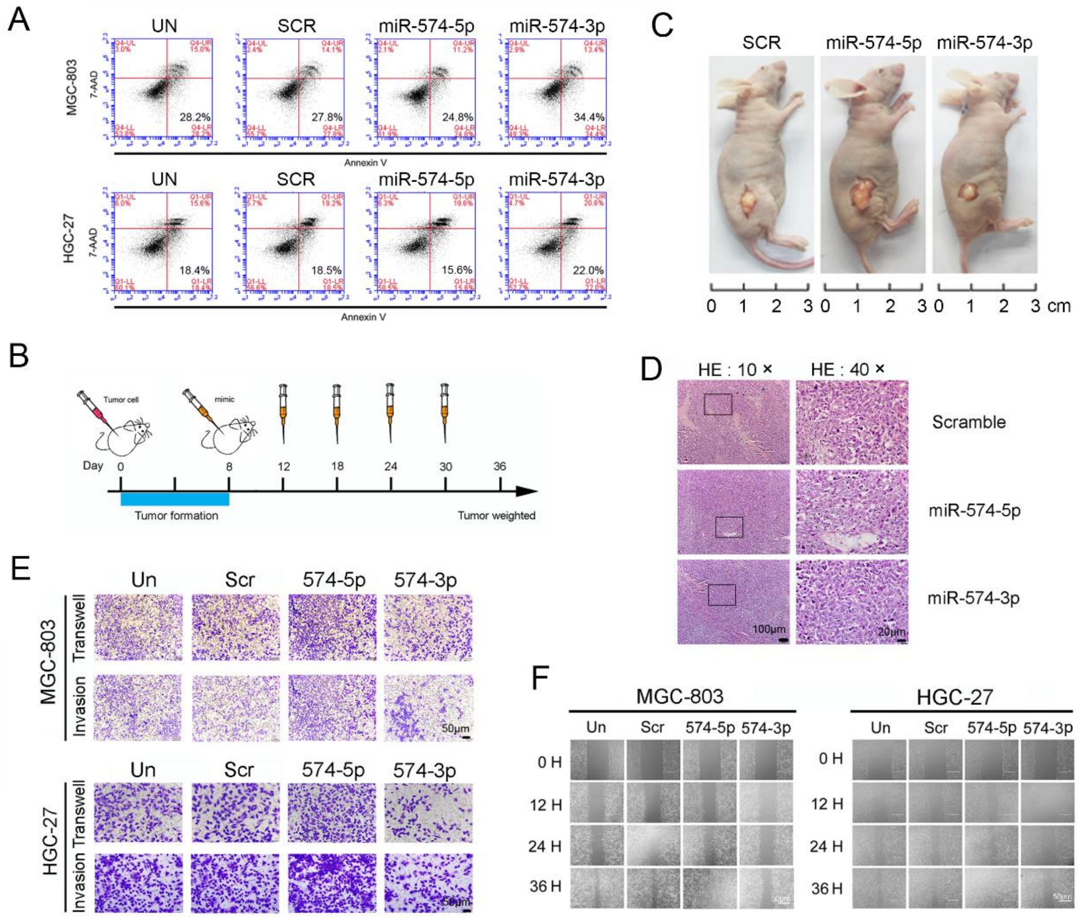

Supplementary figure 1 miR-574-5p and -3p divergently control GC cell growth and metastasis. (A) The effects of miR-574-5p/-3p overexpression on GC cell apoptosis. (B) Diagram illustrating the experimental design of the nude mice xenograft experiment. (C) Photographs of mice with engrafted tumors. (D) H&E staining of tumors from xenograft mice. (E) Representative images of transwell and invasion assay of GC cells treated with scramble or miR-574-5p/-3p mimics. (F) Representative images of wound-healing assay of GC cells treated with scramble or miR-574-5p/-3p mimics.

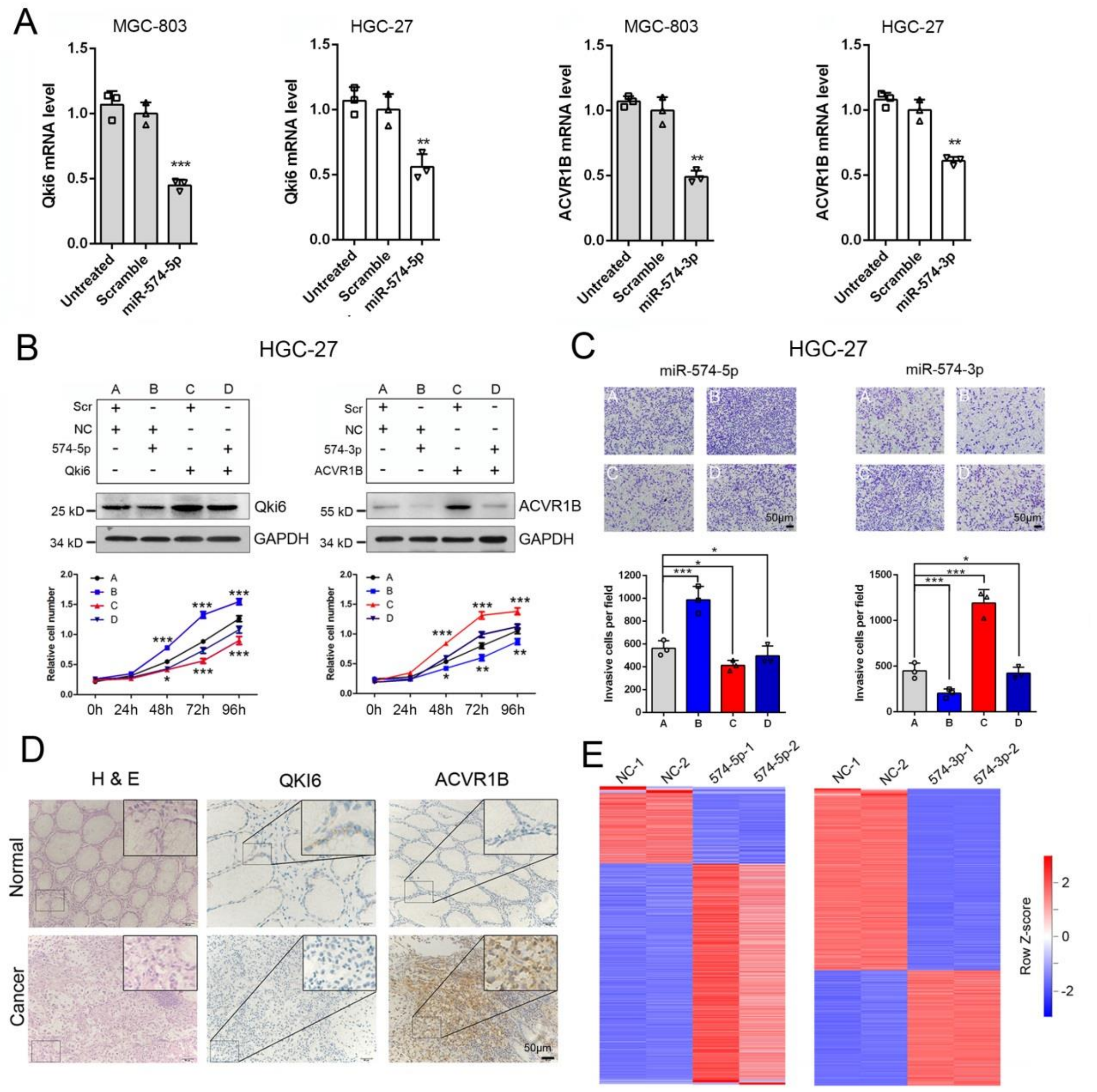

Supplementary figure 2 miR-574-5p/3p targeted endogenous Qki6 and ACVR1B respectively. (A) The relative RNA level of Qki-6 and ACVR1B in GC cells treated with scramble or miR-574-5p/-3p mimics. (B) The protein level of Qki-6 and ACVR1B and cell proliferation rate in HGC-27 cells co-transfected with microRNA mimics and Qki-6 or ACVR1B overexpression. (C) The relative invasive cells of HGC-27 cells in different groups as shown in panel B. (D) Immunohistochemistry analysis of QKI6 and ACVR1B expression in GC patients. (E) Heatmap showing the changes of global gene expression upon miR-574-5p/-3p mimics transfection in MGC-803 cells. Data are shown as means ± s.d.. *P<0.05, **P<0.01, ***P<0.001, Student's t-test.

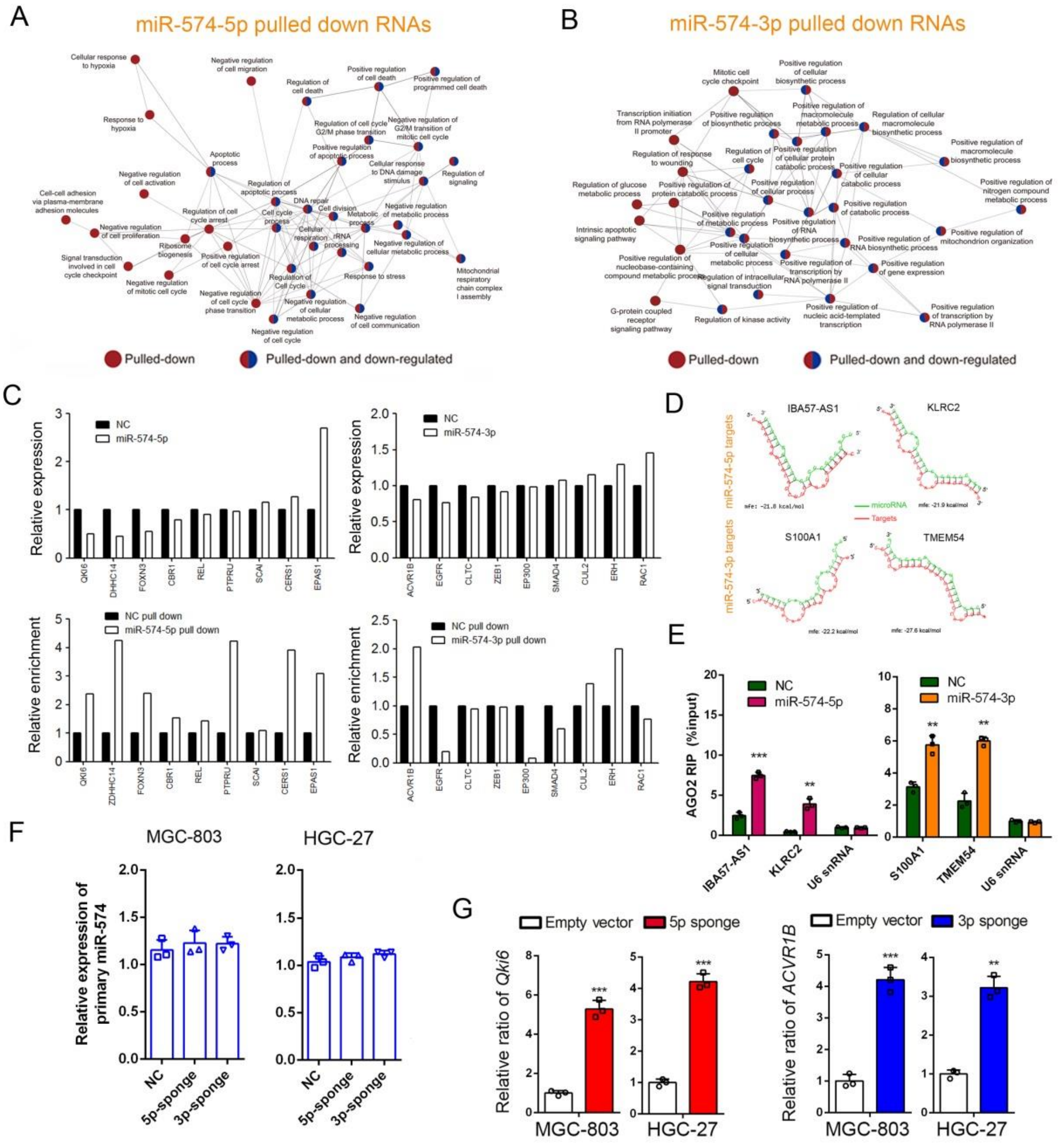

Supplementary figure 3 Identification of the highly complementary targets of miR-574-5p/3p. (A, B) GO and KEGG pathway analysis of miR-574-5p/-3p pulled down targets. (C) The expression change of the reported miR-574 targets in MGC-803 cells with miR-574 overexpression; The relative enrichment of the reported miR-574 targets by biotinylated miR-574 mimics in GC cells. (D) RNAhybrid predicted miR-574-5p or miR-574-3p binding sites in RNA sequence of IBA57-AS1, KLRC2 or S100A1, TMEM54. (E) Ago2 RIP validation of the binding of miRNA and the potential targets. (F) Relative expression of primary-miR-574 in GC cells with artificial miR-574-5p/-3p sponge transfection. (G) RNA level of Qki-6 or ACVR1B in GC cells with overexpression of artificial miR-574-5p/-3p sponges. Data are shown as means ± s.d.. *P<0.05, **P<0.01, ***P<0.001, Student's t-test.

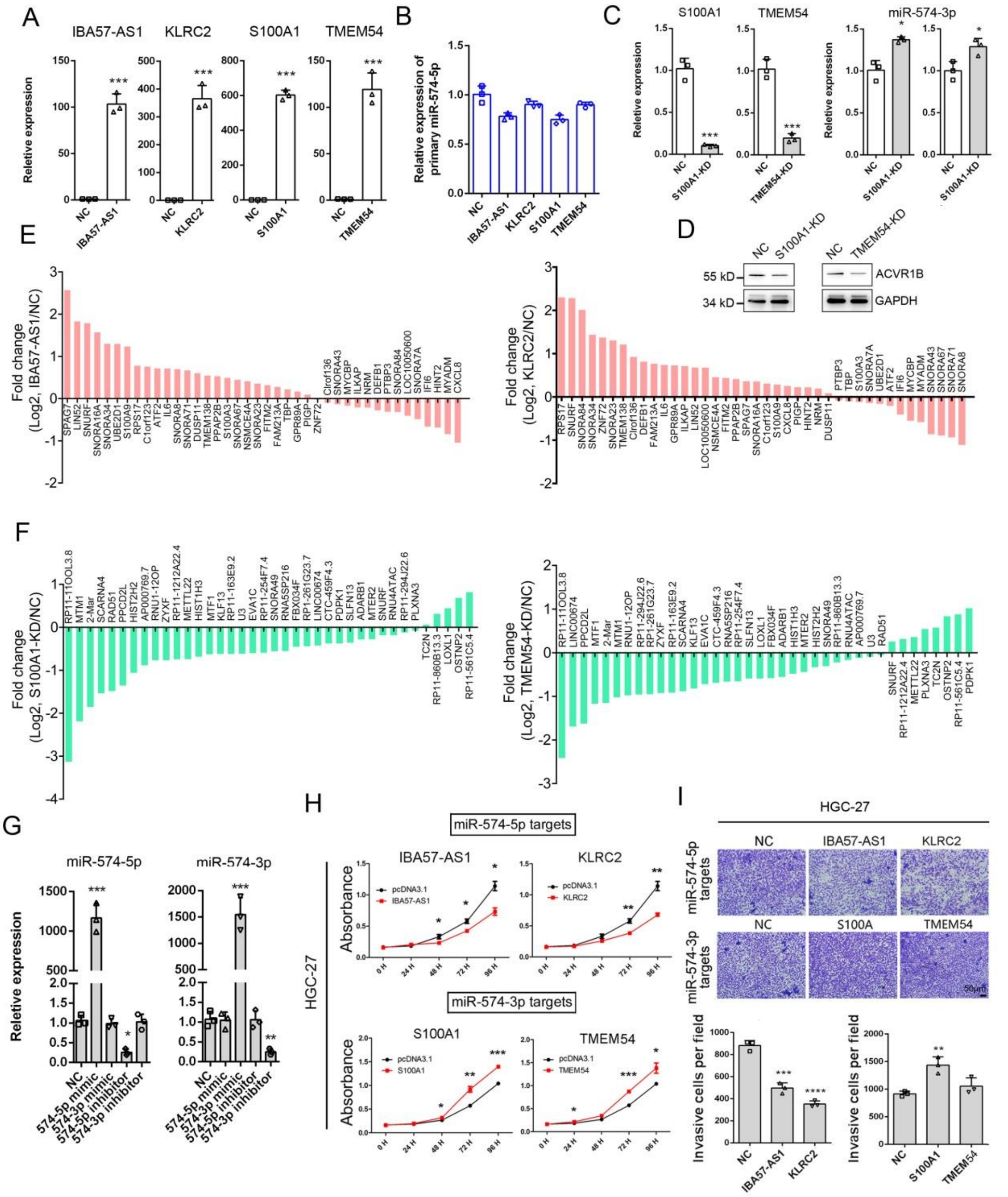

Supplementary figure 4 Potential role of miR-574-5p/-3p targets in GC carcinogenesis. (A) IBA57-AS1, KLRC2, S100A1 and TMEM54 were overexpressed in MGC-803 cells. (B) Relative expression of primary-miR-574 in GC cells with overexpression of IBA57-AS1, KLRC2, S100A1 and TMEM54. (C) Examination of miR-574-3p expression in MGC-803 cells when S100A1 and TMEM54 were suppressed. (D) Protein level of ACVR1B in MGC-803 cells with S100A1 and TMEM54 knock down. (E) The expression change of the potential miR-574-5p targets when IBA57-AS1, KLRC2 was

overexpressed. (F) The expression change of the potential miR-574-3p targets when S100A1, TMEM54 was suppressed. (G) Examination of miR-574-5p and miR-574-3p expression in MGC-803 cells when miR-574-5p/3p was overexpressed or suppressed. (H) The proliferation rate of HGC-27 cells with overexpression of IBA57-AS1, KLRC2, S100A1 and TMEM54. (I) The invasion ability of HGC-27 cells with overexpression of IBA57-AS1, KLRC2, S100A1 and TMEM54. Data are shown as means ± s.d.. *P<0.05, **P<0.01, ***P<0.001, Student's t-test.

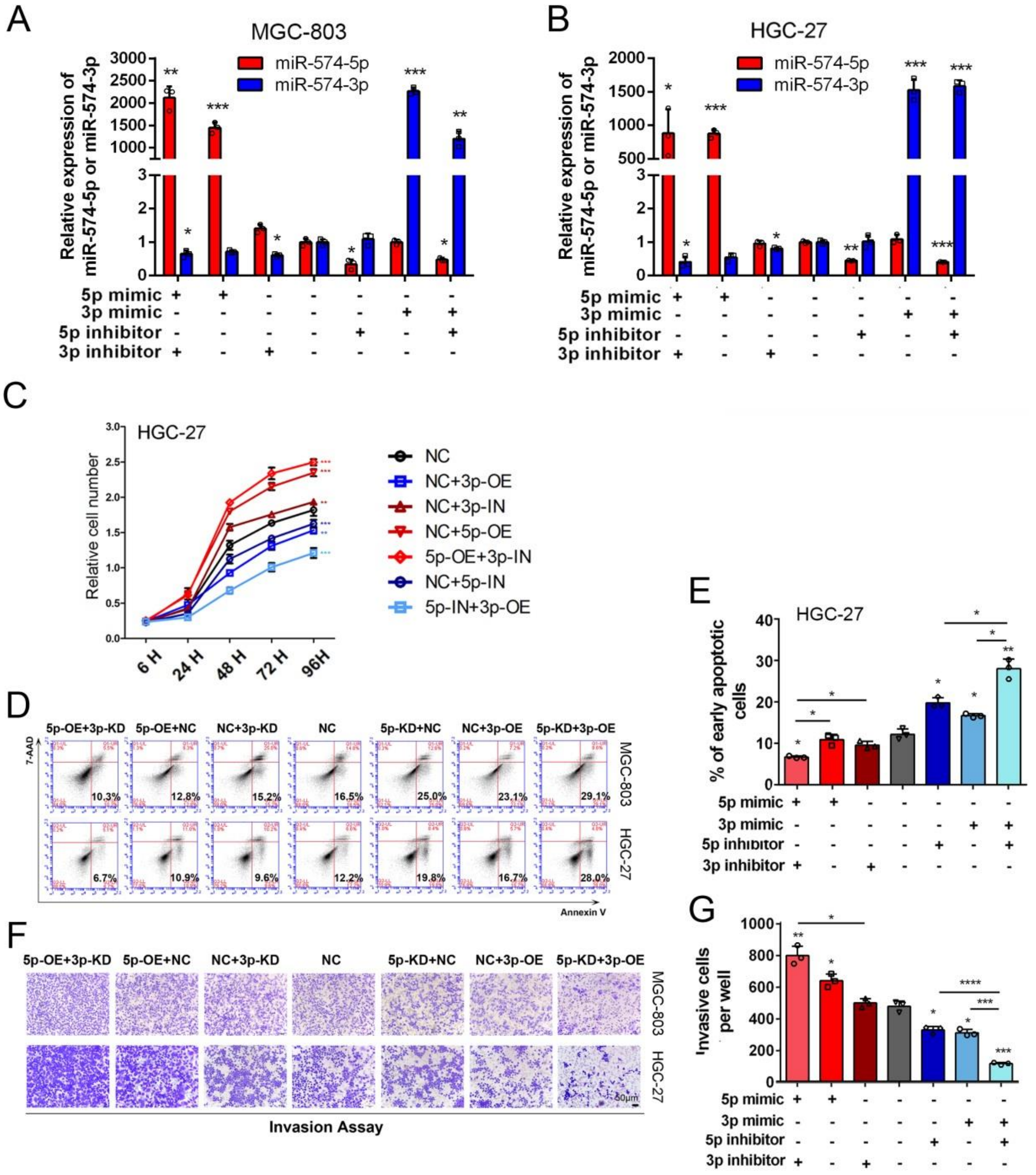

Supplementary figure 5 Arm-imbalance of miR-574 promoted GC progression. MGC-803 and HGC-27 cells were treated with miR-574-5p/3p mimics (5p-OE/3p-OE) and miR-574-5p/3p inhibitors (5p-KD/3p-KD) at multiple combinations to mimic the arm-imbalance. (A, B) The relative expression of miR-574-5p/-3p in MGC-803 (A) and HGC-27 (B) cells. (C) The proliferation rate of HGC-27 cells of the above seven groups. (D, E) Cell apoptosis was analyzed in MGC-803 and HGC-27 cells and the representative result was shown. (F) Representative images of *in vitro* migration assay of MGC-803 and HGC-27 cells. (G) Invasive cell number of HGC-27 cells of the above seven groups. Data are shown as means ± s.d.. *P<0.05, **P<0.01, ***P<0.001, Student's t-test.

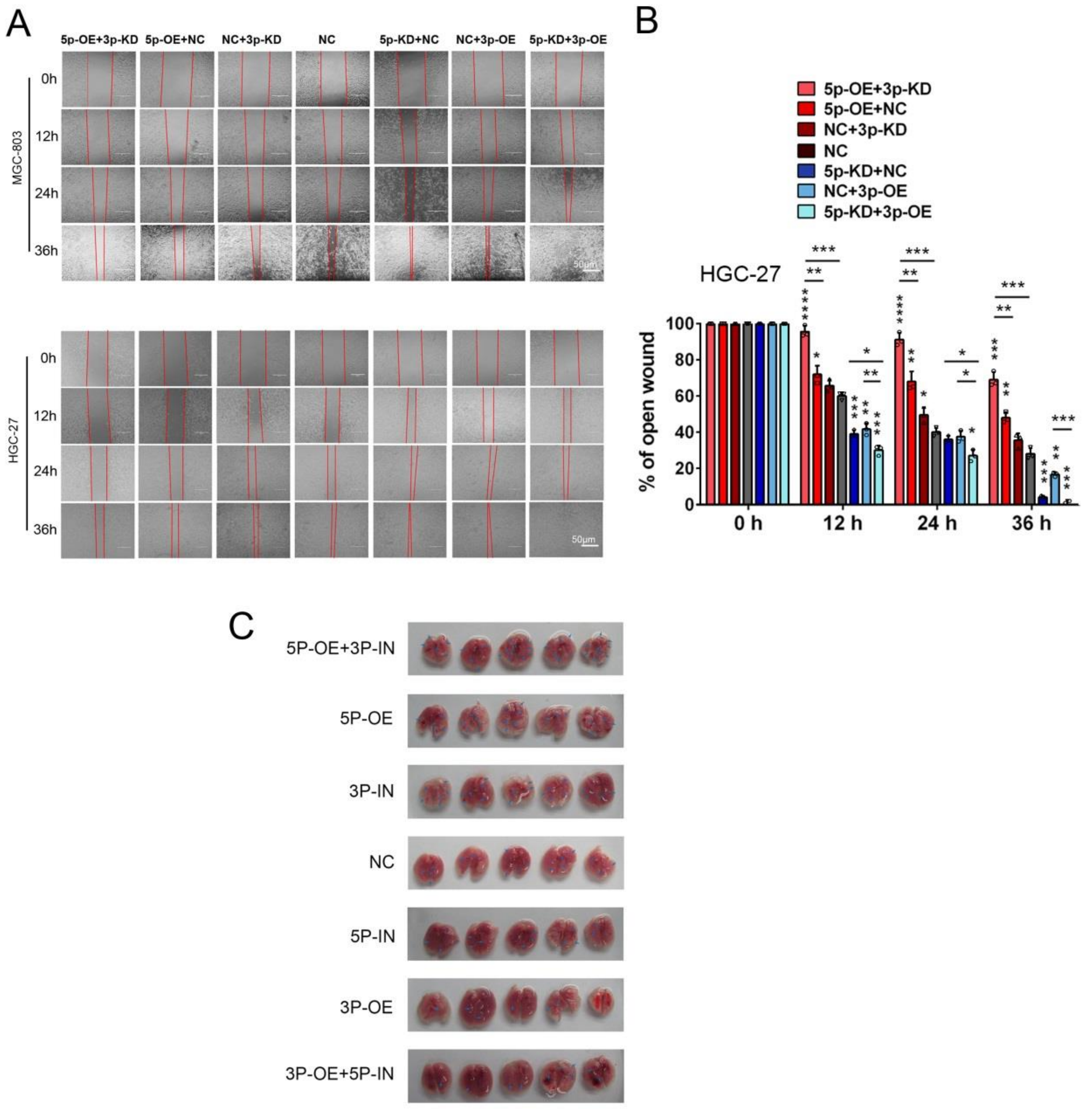

Supplementary figure 6 Arm-imbalance of miR-574 promoted GC progression.

(A) Representative images of wound-healing assay of MGC-803 and HGC-27 cells. (B) Percentage of wound closure of HGC-27 cells of the above seven groups. (C) Images of metastatic nodules formation in lung tissues. Data are shown as means ± s.d.. **P*<0.05, ***P*<0.01, ****P*<0.001, Student's *t*-test.

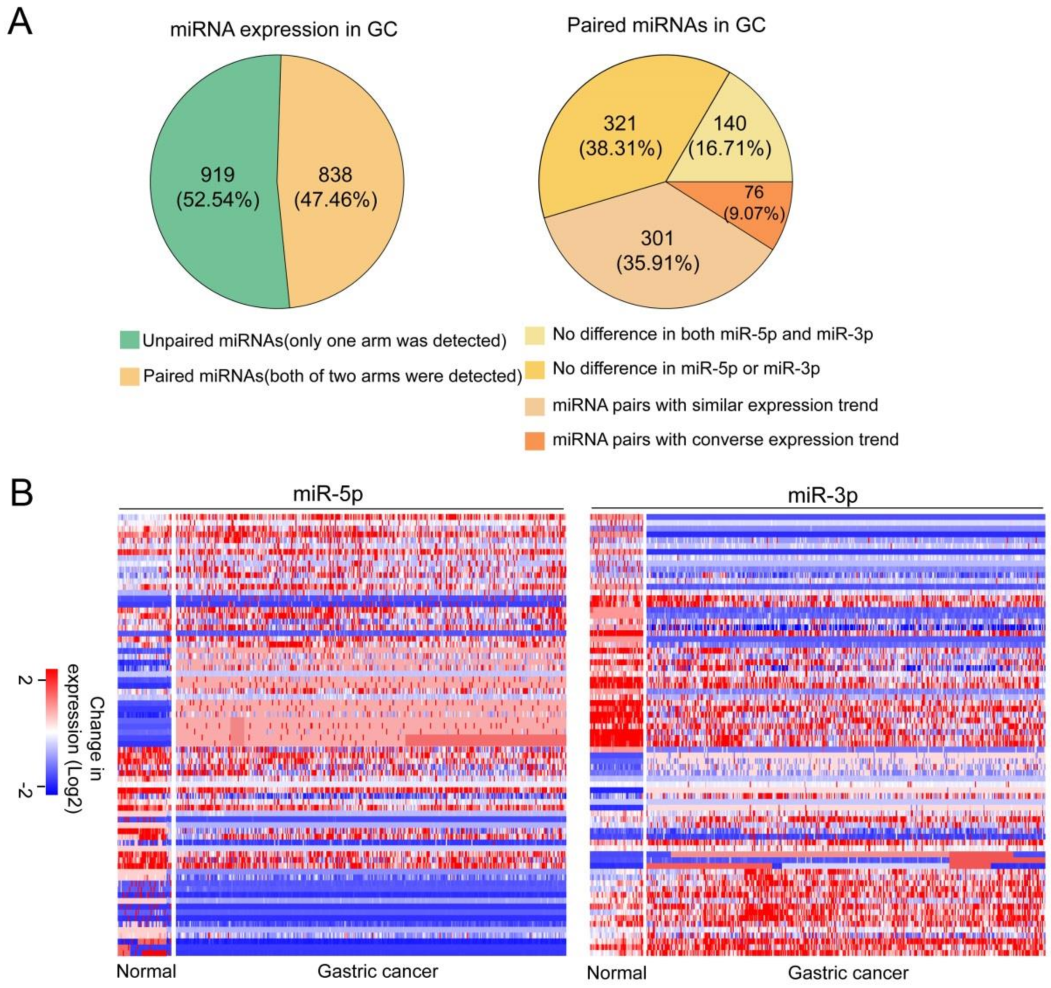

Supplementary figure 7 Global analysis of two arms of a miRNA (miR-5p and miR-3p) in gastric cancer tissues. (A) The expression and variation of miR-5p and miR-3p in gastric cancer tissues. (B) The expression change of miR-5p and miR-3p from the same miRNA precursor in gastric cancer tissues compared with the normal controls.

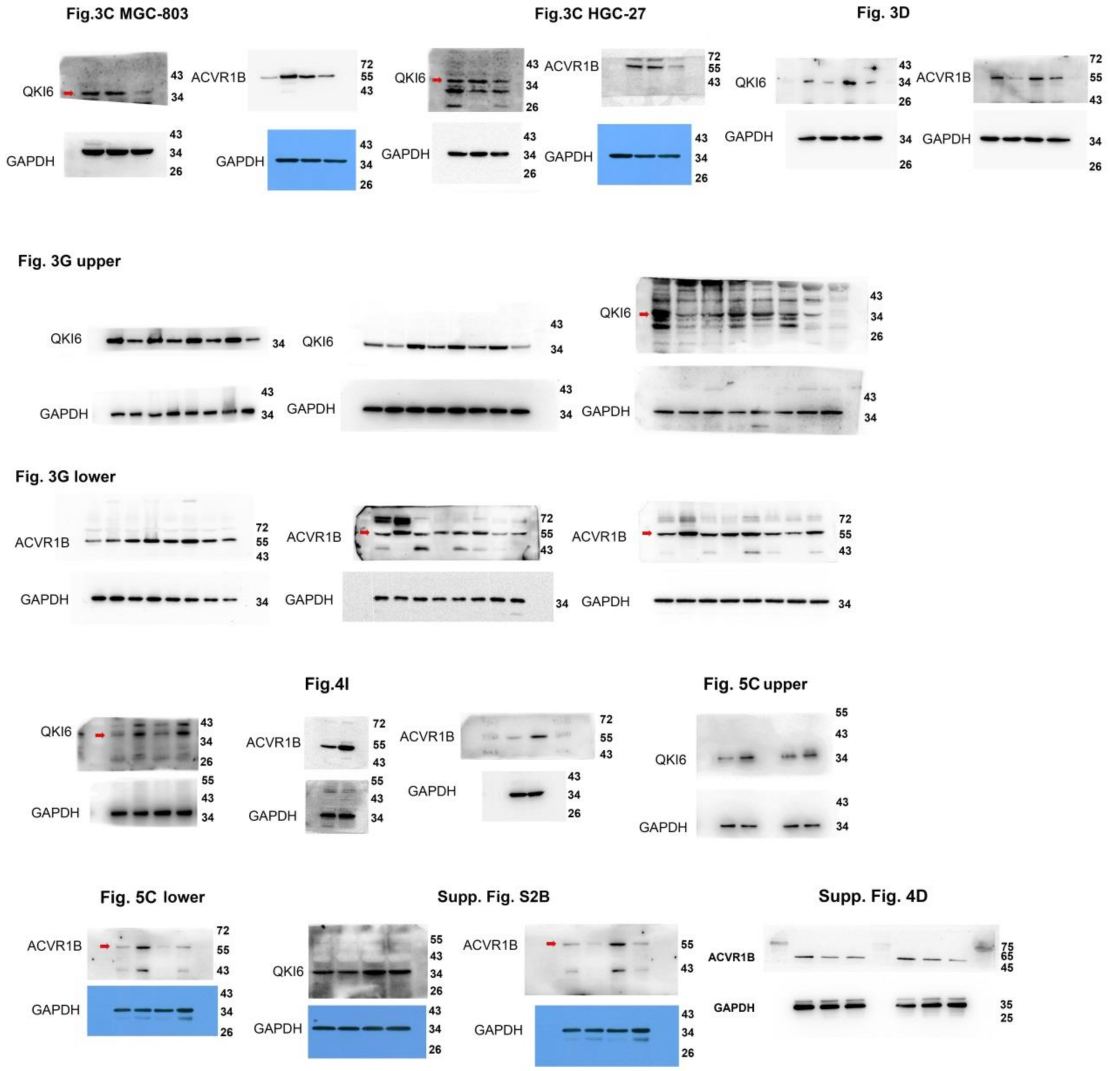

Supplementary figure 8 Uncropped and unprocessed scans of the immunoblots.

Supplementary Tables

Supplementary Table 1. Association Between miR-574-5p Levels in gastric cancer and Clinical Findings

Variables	574-5p log2 C/N<1 (N)	574-5p log2 C/N>1 (N)			X^2	*P*
Gender						
Male	43	81			1.207	0.272
Female	11	32				
Age (years)						
< 60	24	51	0.042	0.593	0.001	0.972
≥60	30	63				
Location						
Upper	22	35			4.496	0.106
Middle	19	22				
Down	9	29				
TNM stage						
I	5	2	0.137	0.092	7.965	0.047
II	17	24				
III	22	62				
IV	8	12				
Perineural invasion						
Negative	34	69	0.097	0.213	0.146	0.702
Positive	19	44				
Tumor thrombus						
Negative	28	66	-0.094	0.231	0.644	0.422
Positive	25	45				

Supplementary Table 2. Association Between miR-574-3p Levels in gastric cancer and Clinical Findings

Variables	574-5p log2 C/N<1 (N)	574-5p log2 C/N>1 (N)	Spearman R	*P*	X^2	*P*
Gender						
Male	81	43			0.001	0.980
Female	28	15				
Age (years)						
< 60	51	23	-0.019	0.806	0.550	0.458
≥60	59	34				
Location						
Upper	36	21	-0.059	0.492	5.343	0.069
Middle	21	20				
Down	29	9				
TNM stage						
I	1	6	-0.311	0.000	27.256	0.000
II	18	23				
III	69	15				
IV	12	8				
Perineural invasion						
Negative	68	35	-0.113	0.147	0.015	0.901
Positive	41	22				
Tumor thrombus						
Negative	68	26	0.126	0.109	39.320	0.000
Positive	16	54				

Supplementary Table 3. Survival analysis.

Variables	Patients (N = 124)	Deaths (%) (n = 73)	MST[a] (months)	Log-Rank P	Adjusted HR	95% CI	P
miR-574-5p (log2 C/N)							
<1	47	19 (40.4)	Not reached	0.009	1.825	1.089-3.058	0.022
>1	77	54 (70.1)	34.467				
miR-574-3p (log2 C/N)							
>1	46	20 (43.5)	Not reached	0.020	1.989	1.177-3.363	0.010
<1	78	53 (67.9)	34.467				
No. of high-risk miRNA							
0	29	10 (34.5)	Not reached	0.010	1		0.013
1	35	19 (54.3)	54		1.577	1.299-5.164	0.244
2	60	44 (73.3)	32		2.590	0.733-3.393	0.007
Age (years)							
<60	50	25 (50.0)	57.93	0.055	1.598	0.985-2.592	0.058
≥60	74	48 (35.1)	33.63				
TNM stages							
I+II	38	6 (20.2)	Not reached	0.000	9.174	3.959-21.258	0.000

III+IV	84	67 (84.2)	25.80				
Nervous invasion							
Absent	72	45 (37.5)	40.80	0.518	0.855	0.530-1.378	0.520
Present	50	27 (46.5)	44.03				
Tumor thrombus							
Absent	73	46 (37.0)	41.46	0.499	1.178	0.732-1.895	0.500
Present	50	27 (46.0)	44.03				

NOTE. [a]MST: Median survival time.

Supplementary Table 4. Primers used in this study.

Primers for real-time RCR of miRNAs (one common reverse primer MiR-anti was used for all miRNAs) (5’-3’)	
miR-574-5p forward	GCGTGAGTGTGTGTGTGTGA
miR-574-3p forward	GCACGCTCATGCACACA
miR-anti	GTGCAGGGTCCGAGGT
U6 forward	CTCGCTTCGGCAGCACATATACT
U6 reverse	ACGCTTCACGAATTTGCGTGTC
Primers for real-time RCR of genes (5’-3’)	
Qki6 forward	GCCCGAAGCTGGTTTAATCTATA
Qki6 reverse	TCGTTGGGAAAGCCATACCTAAT
KLRC2 forward	TAAATGGTTTGGCTTTCAAACA
KLRC2 reverse	TGAAGATCCACACTGGGCTGAT
RUN12 forward	CACGCCTAAAAAGTAGACTGAC
RUN12 reverse	TAACGGAGAAAAGAACGAAAAA
IBAS57-AS1 forward	CAGAGGACAATTTAGGCAGGAA
IBAS57-AS1 reverse	CTCAGCAGGGGGTATGGGACA
RPS17 forward	GCCCAGTAAGAGGTATCTCCAT
RPS17 reverse	TCAATAATCTCCTGATCCAAGG
EAF2 forward	TTGGTGAAGGTGAACAGGTGAC
EAF2 reverse	AAATGCATTCTTTTAAGTAAGG
SOX18 forward	GCCAGGCGGCAGACGAGT
SOX18 reverse	CGTTGTGCAGGTCCGGGTT
S100A1 forward	ACTTCCAGGAGTATGTGGTGCT
S100A1 reverse	TGTGGCTGTCTGCTCAACTGT
NTN4 forward	AGGGAATCGTAGTGGTGGTGT
NTN4 reverse	CATCTGGAGCTGAGAAGGGTCT

TMEM forward	CGGAGAACGTGTTTGCTGTA
TMEM reverse	ATCATGTGGTGGCTGCTTT
IQCH-AS1 forward	CGTGGGTTCTCACTATGTTGC
IQCH-AS1 reverse	CAGTTGGACATGGTGGCTTCT
ACVR1B forward	ATGTGGCTGTGAAAATATTCTCTTCT
ACVR1B reverse	AGTGTTTCATATTAATGGTTTCATCAAG
β-actin forward	CTGGCACCACACCTTCTACA
β-actin reverse	AGCACAGCCTGGATAGCAAC
GAPDH forward	TCAACGACCACTTTGTCAAGCTCAGCT
GAPDH reverse	GGTGGTCCAGGGGTCTTACT
Primers for constructs in dual luciferase reporter experiments (5’-3’)	
Qki6 3'UTR forward	TGCTCTAGACTTTGTTAAGTAATCCACACTC
Qki6 3'UTR reverse	ACTGGCCGGCCAACGGTTGTCCCATAGTCTTAA
ACVR1B 3'UTR forward	CGGGATCCCGTCTAACTGCTCCCTCTCT
ACVR1B 3'UTR reverse	GCTCTAGAGCGTGTCTGTCTCAAACCCA
Primers for constructs in gene overexpression (5’-3’)	
KLRC2 forward	CGGGATCCGATGAATAAACAAAGAGGAACCT
KLRC2 reverse	GCTCTAGATTTCTGGATAGCTTTATTGAAGT
IBAS57-AS1 forward	CGGGATCCACGTGTGGCTGTTGTGTCAT
IBAS57-AS1 reverse	GCTCTAGAGACCTCTGCTTGCTGGGACT
S100A1 forward	CGGGATCCATGGGCTCTGAGCTGGAG
S100A1 reverse	GCTCTAGATCAACTGTTCTCCCAGAA
TMEM54 forward	CGGGATCCAAGTGGGCGAGCGCGACG
TMEM54 reverse	GCTCTAGATCAGAGGGTCAGAGGCTCA

Primers for reverse transcription (RT) of mRNAs and microRNAs (5’-3’)	
mRNA RT	TTTTTTTTTTTTTTTTTT
574-5p-RT	GTCGTATCCAGTGCAGGGTCCGAGGTATTCGCACTGGATAC GACACACAC
574-3p-RT	GTCGTATCCAGTGCAGGGTCCGAGGTATTCGCACTGGATAC GACTGTGGG
u6-RT	AAAATATGGAACGCTTCACGAATTTG
Sequences for mimics in microRNA pull-down experiments (5’-3’)	
miR-574-5p	UGAGUGUGUGUGUGUGAGUGUGU
miR-574-3p	CACGCUCAUGCACACACCCACA
miR-574-5p-Mut	UGCUCUGACUUGUGUGAGUGUGU
miR-574-3p-Mut	CAUAUCAGCUCACACACCCACA
NC-mimic	UUUGUACUACACAAAAGUACUG
