# Supplementary material for: microRNA arm-imbalance in part from complementary targets mediated decay promotes gastric cancer progression
Source: Nat Commun. 2019 Sep 27;10:4397. doi: 10.1038/s41467-019-12292-5 (PMC6764945; doi:10.1038/s41467-019-12292-5)
Supplement: Supplementary file 3 — Description of Additional Supplementary Files [file 41467_2019_12292_MOESM3_ESM.pdf]

Description of Additional Supplementary Files

File Name: Supplementary Data 1
Description: Global gene expression analysis in MGC-803 cells with miR-574 overexpression.

File Name: Supplementary Data 2
Description: RNA-sequencing of biotinylated miRNA pull-down.

File Name: Supplementary Data 3
Description: miR-574-5p/-3p isoform analysis in MGC-803 with IBA57-AS1, KLRC2, S100A1 and TMEM54 overexpression.

File Name: Supplementary Data 4
Description: Global gene expression analysis in MGC-803 cells with IBA57-AS1, KLRC2 overexpression or S100A1, TMEM54 knock down.

File Name: Supplementary Data 5
Description: miRNA pairs showed converse expression change in gastric cancer compared with normal controls.
